# Color-Changing Reflection Hologram for Quality Assurance of Therapeutic Ultrasound Systems

**DOI:** 10.1021/acsami.3c06139

**Published:** 2023-07-21

**Authors:** Tatsiana Mikulchyk, John Walsh, Jacinta Browne, Izabela Naydenova, Dervil Cody

**Affiliations:** † Centre for Industrial and Engineering Optics, School of Physics, Clinical and Optometric Sciences, Technological University Dublin, Grangegorman Campus, Central Quad, Grangegorman Lower, D07 ADY7 Dublin, Ireland; ‡ School of Art and Design, Technological University Dublin, Grangegorman Campus, Grangegorman Lower, D07 ADY7 Dublin, Ireland; § Department of Radiology, Mayo Clinic, Rochester, Minnesota 55905, United States

**Keywords:** therapeutic ultrasound, physiotherapy, quality assurance, holography, photopolymer, grating

## Abstract

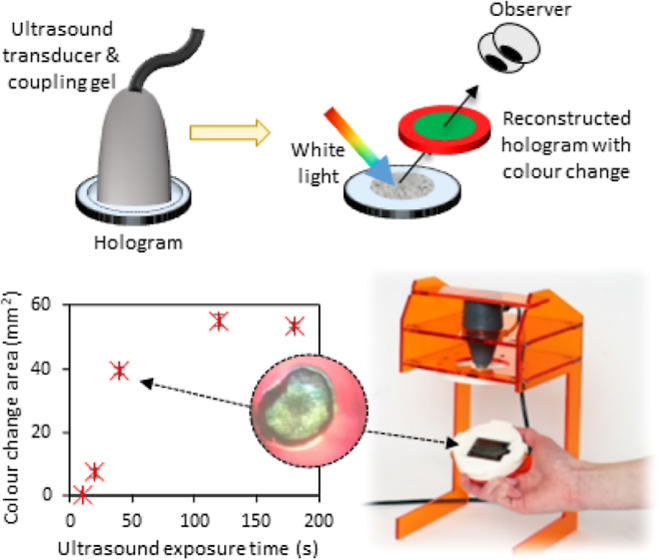

The acoustic output of clinical therapeutic ultrasound equipment requires regular quality assurance (QA) testing to ensure the safety and efficacy of the treatment and that any potentially harmful deviations from the expected output power density are detected as soon as possible. A hologram, consisting of a reflection grating fabricated in an acrylate photopolymer film, has been developed to produce an immediate, visible, and permanent change in the color of the reconstructed hologram from red to green in response to incident ultrasound energy. The influence of the therapeutic ultrasound insonation parameters (exposure time, ultrasound power density, and proximity to the point of maximum acoustic pressure) on the hologram’s response has been investigated for two types of therapeutic ultrasound systems: a sonoporation system and an ultrasound physiotherapy system. Findings show that, above a switching temperature of 45 °C, the ultrasound-induced temperature rise produces a structural change in the hologram, which manifests as a visible color change. The area of the color change region correlates with the ultrasound exposure conditions. The suitability of the hologram as a simple and quick QA test tool for therapeutic ultrasound systems has been demonstrated. A prototype ultrasound testing unit which facilitates user-friendly, reproducible testing of the holograms in a clinical setting is also reported.

## 1 Introduction

Ultrasound is a form of mechanical energy which propagates in media as a longitudinal wave. Ultrasound-based medical therapies utilize the localized thermal and nonthermal therapeutic effects produced by the interaction between ultrasonic waves and biological tissue. Due to the advantageous properties of being both nonionizing and minimally invasive, ultrasound-based therapies have been widely applied in medicine. The popularity of these therapies is growing; the therapeutic ultrasound market was valued at $1.93 billion in 2021, and a compound annual growth rate of 8.5% is forecast through to 2030.^1^

The properties of the ultrasound waves used in therapeutic devices and equipment vary significantly and are summarized in Table 1. Lower power therapeutic ultrasound systems which emit lower amplitude waves (<1 MPa) via a transducer head coupled in direct contact with the skin are widely used in physiotherapy to treat muscular pain and tendinitis in both human^2^ and animal patients.^3^ The reported thermal effects of ultrasound include increase in the extensibility of collagen-rich scar tissues, tendons and joints, relief of pain and muscular spasm, stimulation of cells by upregulation of signalling molecules, activation of immune cells, remodelling of scars, and accelerating bone fracture healing. For these effects to be achieved, a temperature increase of 8–10 °C (actual tissue temperature 45–47 °C) is typically required.^4^ Sonoporation is used in medical research applications such as gene therapy and drug delivery due to the production of thermal and nonthermal effects in tissue.^5^ Achievement of these effects typically requires a temperature increase of 10–16 °C (actual tissue temperature 47–53 °C).^6^ High-intensity focused ultrasound (HIFU) is an emerging and exciting therapy in which masses such as tumors and uterine fibroids are thermally ablated by highly focused ultrasound waves emitted from a HIFU transducer.^7^ During HIFU, live tissue is heated beyond the threshold for protein denaturation (57–60 °C) for a few seconds as happens during focused ultrasound (FUS) ablation, and coagulation necrosis occurs.^8^ Each of the applications described above induce supraphysiological temperatures (>40 °C) in biological tissues causing changes at the molecular, cellular, and structural level, with corresponding changes in tissue function. Achievement of the required temperature increase in the target tissue, and thus the desired treatment outcome, depends on the acoustic output of the ultrasound therapy system.

**Table 1:** Summary of the Properties of Ultrasound Waves as Used in Different Therapies

	typical power (W)	typical *I*_spta_ (W/cm^2^)	temperature range produced in tissue (°C)
physiotherapy	5	1.5	45–47^4^
sonoporation	5	5	47–53^6^
HIFU/FUS	200	1000	57–60^8^

Standards published by the International Electrotechnical Commission (IEC) provide recommendations for the acoustic field parameters, as well as guidance for performance evaluation of the different ultrasound therapy devices, depending on the application.^9−12^ Adherence to these standards is essential for patient safety and treatment efficacy; excessive acoustic pressures and prolonged treatment exposure times have the potential to result in damage to surrounding tissue due to unwanted temperature elevation, while insufficient pressure and treatment exposure time risks failed or ineffective clinical treatments. Despite the existence of these standards, a significant and troubling difference between the expected and measured outputs of physiotherapy systems in clinical use has been routinely reported.^13−17^ One 2008 Australian study^13^ reported that 59% of 64 physiotherapy systems tested were producing insufficient or excessive acoustic energy. While the physiotherapy systems initially conform to technical standards, wear and tear over time, particularly to the transducer face, may degrade their performance. The variation in system output can be partially explained by low awareness of the importance of regular quality assurance (QA) testing; a 2010 study by Ferrari et al. showed that while the surveyed professionals were not concerned with equipment calibration, only 32.3% of the same professionals’ equipment was in accordance with the norms for power and effective radiation area.^16^ Moreover, the guidelines for performance evaluation of physiotherapy systems outlined in IEC 61689^10^ require a scanning hydrophone or a radiation force balance apparatus for measurement of the acoustic field and output power, respectively. These techniques are expensive (likely exceeding the purchase price of the physiotherapy system itself), time-consuming and complex, requiring laboratory conditions which are not achievable in a busy clinical setting.

It is evident that operators of ultrasound therapy devices would benefit from the availability of alternative methods to perform simple, quick verification of the ultrasound system operating performance on a regular basis or indeed daily QA prior to treatments. Indeed, the need and justification for use of periodic qualitative QA methods to be used in conjunction with less frequent, robust quantitative assessment of the device performance were established in IEC TS 62462.^18^

Research efforts toward the development of qualitative analysis methods for ultrasound systems have centered largely on thermochromic materials. In 1971, Cook and Werchan discovered the applicability of thermochromic liquid crystals in spatial mapping of an incident ultrasound field via a color change which corresponds to the localized absorption-induced temperature rise.^19^ Martin and Fernandez further developed the technique of Cook and Werchan to the point of producing stable intensity distributions in a commercially available thermochromic tile; however, their experiment still required a water tank environment.^20^ More recently, the development of benchtop thermochromic-based test devices for QA of physiotherapy ultrasound transducers has been reported. Butterworth et al. report a three-layer thermochromic tile manufactured by Acoustic Polymers Ltd which undergoes a partially reversible change in color at a switching temperature (i.e., the temperature at which the color change commences) of 30 °C.^21^ The suitability of this device as a benchtop QA tool for qualitative measurements of nine clinically used physiotherapy transducers was successfully shown by Žauhar et al.^22^ A thermochromic pigment-doped silicone phantom which has a switching temperature of 45 °C is described by Costa et al.^23^ The repeatable production and correlation of ultrasound energy-induced heating patterns in the thermochromic phantom with the ultrasound beam profile demonstrates the capability of such simplistic test devices for qualitative assessment of ultrasound system performance on an ongoing basis.

**Figure 1:**
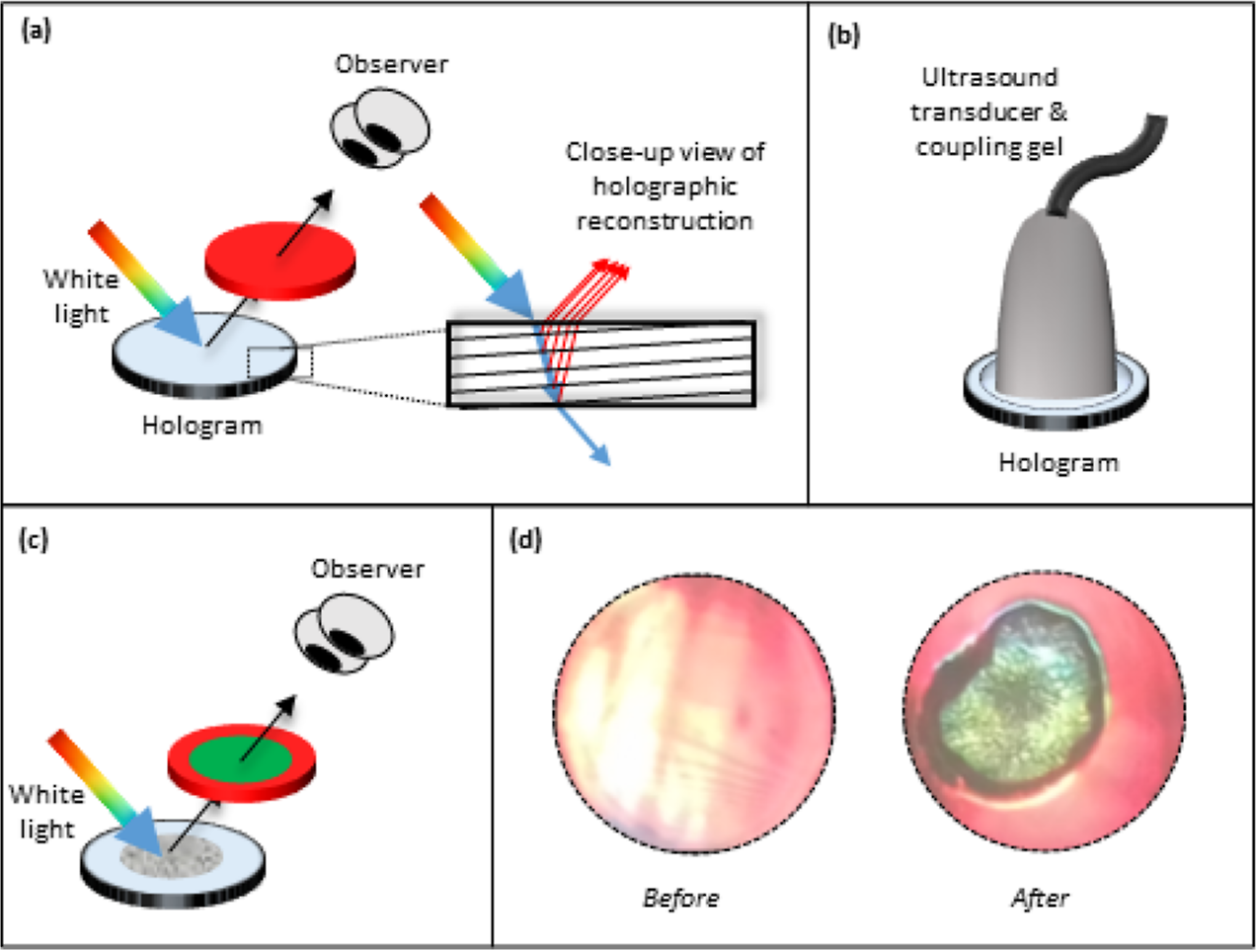
Principle of operation of the reflection hologram for ultrasound QA. (a) Hologram is reconstructed using white light, and a red reflection grating is visible at the reconstruction angle. (b) Hologram is exposed to therapeutic ultrasound via a transducer (and coupling gel); (c) region of color change from red to green is visible on the reconstructed hologram following ultrasound exposure. (d) Photograph of the hologram, reconstructed with white light, before and after ultrasound exposure.

Here, we present a hologram that can be used for quick qualitative or semi-quantitative assessment of the performance of a range of therapeutic ultrasound systems. The use of holographic structures for detection and sensing of an array of chemical analytes, biological species, and environmental stressors has been widely reported in the literature.^24−32^ This is due to the potential of holographic sensors for high sensitivity, fast response time, high selectivity via careful material design, low cost, and suitability for mass production.^33,34^ In this instance, the hologram consists of a reflection grating inscribed in a photosensitive polymer film; the development and optimization of the low-toxicity photopolymer composition for the fabrication of bright, stable reflection holograms have previously been reported.^35^ The color of the reconstructed hologram undergoes a permanent change from red to green after exposure to a specific dose of ultrasound energy (Figure 1). Like the work of Costa et al., the dimensions of the area of color change can be quantified and compared to a reference measurement acquired during an initial system setup. Unlike the thermochromic-based devices discussed above which undergo a nonstructural color change and respond to change in temperature only, the color change of the hologram is produced by the permanent distortion of the holographic structure inside the film due to both elevated temperature and alternating pressure. While more complex, the specificity of the response of the holographic sensor is advantageous, as it prevents interference from other sources of heat that could interfere with the interpretation of the sensor readout. The holographic nature provides an innovative and interesting dimension for therapeutic ultrasound QA and pre-treatment checks that has not been reported in the literature thus far.

For the proof-of-concept testing reported here, two different types of ultrasound therapy systems were selected: a sonoporation system (Sonidel SP100) and a physiotherapy system (gbo Medizintechnik Sonostat 133). Several of the aforementioned studies outlining novel thermochromic test devices for ultrasound transducers^21−23^ highlight the importance of consistent ultrasound transducer positioning and coupling in ensuring accurate and reproducible results. Issues surrounding stability and variability in externally applied pressure to the transducer are reported to influence the QA test results.^22,23^ The need for specialist setups to facilitate accurate and reproducible positioning of the ultrasound transducer at a fixed distance from the test device is therefore crucial. We present an initial prototype of an innovative and user-friendly ultrasound testing unit which meets these requirements. The testing unit was custom designed for use in conjunction with the Sonidel SP100 Sonoporator system, to provide for accurate and consistent positioning of the hologram in relation to the sonoporator transducer, therefore improving repeatability of the procedure and ease of use. However, the testing unit design can be modified for different therapeutic ultrasound units.

## 2 Materials and Methods

### 2.1 Hologram Fabrication

#### 2.1.1 Photopolymer Film Preparation

The photopolymer composition used to fabricate the reflection gratings is outlined in Table 2. The role of the individual photopolymer components during holographic recording has been reported in detail elsewhere.^27,35^ Following thorough mixing of the photopolymer components, 0.7 mL of the photopolymer solution was deposited onto levelled glass slides (75 × 25 mm) and dried for 24 h in a dark room at an environmental temperature of 21 ± 2 °C and a relative humidity of 30 ± 5%. The dry film thickness was measured to be 160 ± 5 μm.

**Table 2:**
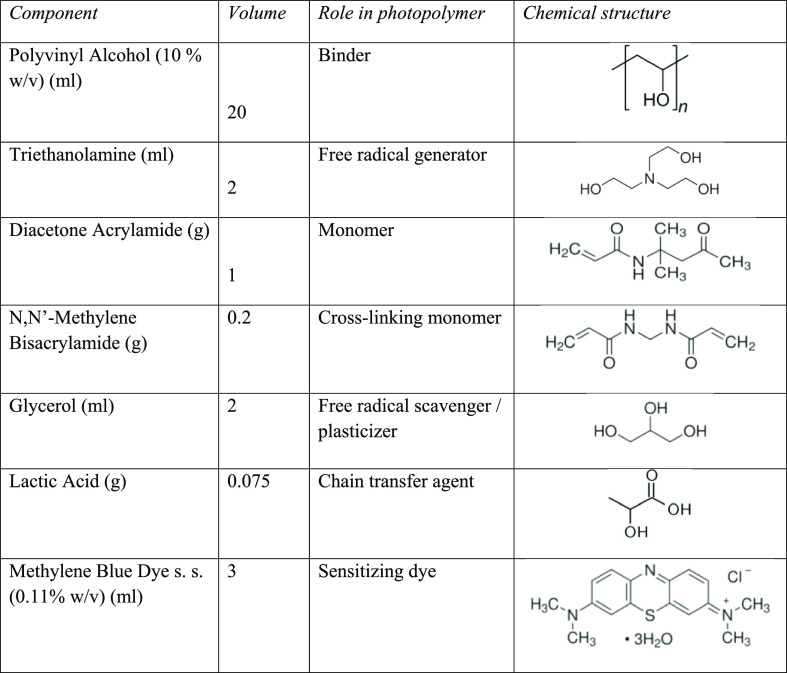
Chemical Composition of Reflection Mode Photopolymer

#### 2.1.2 Holographic Recording of Reflection Gratings

**Figure 2:**
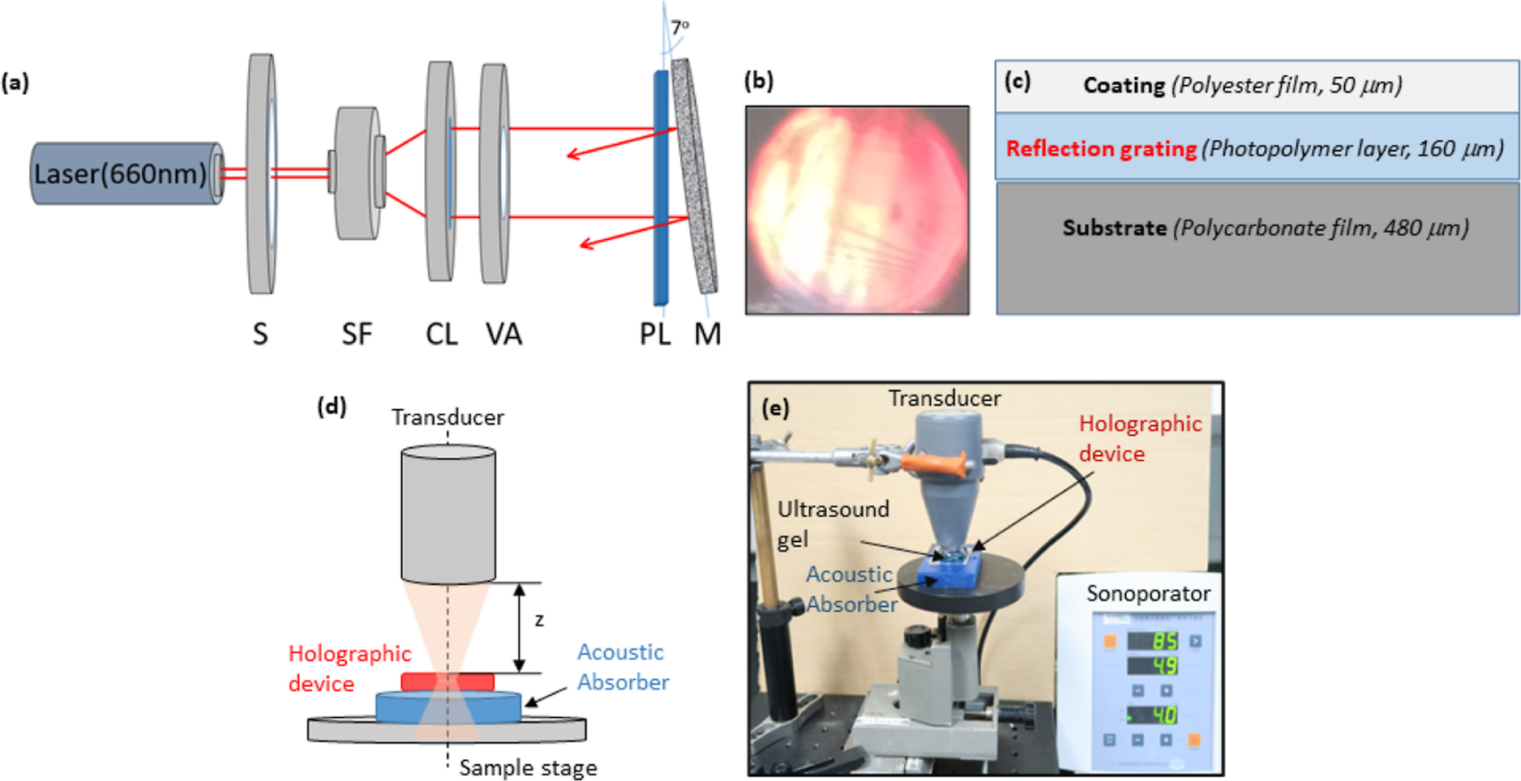
(a) Denisyuk holographic recording setup: S: shutter; SF: spatial filter; CL: collimator; VA: variable aperture; PL: photopolymer layer; M: mirror. (b) Photograph of reflection grating taken in room lighting. (c) Schematic of the final holographic device structure. (d) Schematic and (e) labelled photograph of sonoporator testing setup.

A 660 nm Cobolt Flamenco 500 laser was used to record the reflection gratings in the photopolymer films using a Denisyuk holographic geometry, shown in Figure 2a. Briefly, the single 660 nm beam is spatially filtered, collimated, and directed at normal incidence on to the front surface of the photosensitive film; this is the reference beam. The partially transmitted beam is then redirected back on to the photosensitive film using a mirror, thereby creating the object beam. In order to record the reflection grating, the photosensitive film is positioned at the point of interference of the object and reference beams. The angle of the mirror relative to the photosensitive film was controlled using a high-performance precision rotation stage (Newport, M-481-A) and set to 7°, which corresponds to a spatial frequency of ∼4500 lines/mm. The output power of the laser was directly controlled by the accompanying Cobolt Flamenco software. All beam intensity measurements were made using a Newport 1830-C optical power meter. The diffraction efficiency, η, of the recorded reflection gratings was calculated using
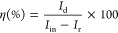
1where *I*_in_ is the incident probe beam intensity and *I*_d_ and *I*_r_ are the intensities of the diffracted and reflected (Fresnel reflection at the front surface) beams, respectively. A low intensity (∼1 mW/cm^2^) 660 nm beam was used to measure η directly after holographic recording, at which point the photosensitive films are already completely bleached. Using the optimized holographic recording conditions (*I*_in_ = 10 mW/cm^2^, recording time *t* = 100 s), reflection gratings with η = 30% were consistently obtained. The gratings are visibly bright, as shown in Figure 2b, and are approximately 2 cm in diameter. Depending on the profile and dimensions of the ultrasound beam, larger or smaller gratings may be necessary; this can be easily achieved by varying the diameter of the incident recording beam. The gratings were then peeled from the glass substrate and transferred to a polycarbonate substrate (480 μm thick) prior to ultrasound testing (Figure 2c). While glass provides the stability required for recording of reflection holograms in a small-scale laboratory setting, the advantages of plastic substrates are numerous: flexibility, greater versatility, lower cost per device, and suitability for large-scale production.^34^ As the photopolymer film itself is hydrophilic and readily absorbs moisture, the gratings were then coated with a polyester film (50 μm thick), as shown in Figure 2c, to prevent unwanted interaction of the holographic gratings with the ultrasound coupling media.

### 2.2 Ultrasound Exposure of Holograms

#### 2.2.1 Sonoporation System

The Sonidel SP100 (Sonidel Limited, Ireland) is a sonoporator system which was designed to induce localized cavitation effects within a medium. The 1 MHz transducer was designed with a standard columnar ultrasound beam profile. The acoustic field of the SP100 was mapped using a Precision Acoustics 1 mm needle hydrophone (Precision Acoustics, UK) which consisted of a hypodermic needle with a piezoelectric polymer across its tip. When placed in an acoustic field, a voltage was generated across the tip as a function of the pressure of the sound wave. Measurements were taken along the beam alignment axis (center of the beam moving away from the ultrasound transducer) of the spatial peak temporal average acoustic pressure along the center of the *z*-axis from the transducer face. The acoustic power (*W*) produced by the transducer as a function of set power was measured using a Precision Acoustics Radiation Force Balance (Precision Acoustics, UK). The transducer face was submerged in degassed water 10 mm above an acoustic absorber target (Precision Acoustics, UK, Aptflex F28P, insertion loss approximated by 20 dB/cm/MHz). The displacement that the transducer produced on the radiation force balance was recorded in milligrams and converted to a force (*N*) value by multiplying the displacement (mass) by acceleration due to gravity, *g*. Finally, the measured power (*W*) was determined by multiplying the force by the speed of sound in water at the measured temperature of the water. The results of the hydrophone and radiation force balance measurements are presented in Section 3.1.

The following procedure was used to characterize the response of the holograms to the sonoporator ultrasound output. The hologram was placed on an *xyz* positioning stage. A 10 mm thick sheet of the polyurethane-based acoustic absorber (Precision Acoustics, UK, Aptflex F48, insertion loss >60 dB for all frequencies >300 kHz) was positioned between the hologram and the stage to prevent undue heating. The sonoporator transducer was then positioned at normal incidence to the hologram surface, as shown in Figure 2d,e. Room-temperature ultrasound coupling gel was liberally used to ensure acoustic coupling at the different interfaces and prevent air bubbles. Fresh coupling gel was used for each ultrasound exposure.

The Sonidel SP100 sonoporator output is controlled by three parameters: ultrasound power density (up to 5 W/cm^2^), duty cycle (up to 100%, i.e., continuous mode), and exposure time. The influence of these output parameters on the response of the holograms was systematically investigated. The distance, *z*, between the hologram and the transducer face was also systematically varied to facilitate mapping of the ultrasound beam profile.

#### 2.2.2 Physiotherapy System

The gbo Medizintechnik Sonostat 133 is a diverging beam therapeutic ultrasound unit routinely used by physiotherapists with a transducer face size of 2.5 cm^2^. The following procedure was used to characterize the response of the holograms to the physiotherapy system ultrasound output. The hologram was placed on a layer of polyurethane-based acoustic absorber in a plastic container containing 6 mL of distilled water. The 2.5 cm^2^ transducer face was placed in full contact with the hologram surface. The Sonostat parameters were set as follows: ultrasound power density—3 W/cm^2^; pulse duty cycle—100%; output frequency—1 MHz. The influence of the exposure time on the holograms was investigated by measuring the surface temperature and analysing the dimensions of the area of visible change in the appearance of the hologram.

### 2.3 Hologram Characterization

#### 2.3.1 Phase Contrast Microscopy

A phase contrast microscope (Olympus BX51) was used to acquire high-contrast images of the internal structure of the transparent holograms before and after ultrasound exposure. Filter Ph1 and magnification ×4 and ×10 were used for all image acquisitions.

#### 2.3.2 Temperature Measurements

Two methods were used to calibrate and model the behavior of the hologram to ultrasound exposure. The surface temperature of the holograms during ultrasound exposure was monitored locally using a wire thermocouple (TC) placed at the center of the device surface and aligning with the center of the ultrasound beam. A FLIR i7 infrared camera was also used to capture infrared (IR) thermographic images, or temperature maps, of the devices’ surface before and after ultrasound exposure. Both methods were used in this work to calibrate and model the trends in the hologram response to increasing temperature. A comparative study of the temperature of the hologram surface following ultrasound exposure by both the sonoporation and physiotherapy systems is presented in Section 3.2. For this study, the highest power density settings were selected for both units: 5 and 3 W/cm^2^ for the sonoporation and physiotherapy systems, respectively.

#### 2.3.3 Measurement of Area of Color Change

Following each ultrasound exposure, the holograms were placed on a sheet of graph paper with a grid dimension of 7 mm × 7 mm. Photographs of the holograms were obtained, and the area of the opaque region formed on each hologram was estimated using ImageJ 1.53k software. Briefly, the boundary of the opaque area was delineated. The area of the opaque region was then estimated in pixels using the measure function and converted to mm^2^ via comparison with the area of a single 7 mm × 7 mm grid square, as a reference value. Each measurement of area was repeated in triplicate to yield an average and standard deviation value.

### 2.4 Prototype Ultrasound Testing Unit

**Figure 3:**
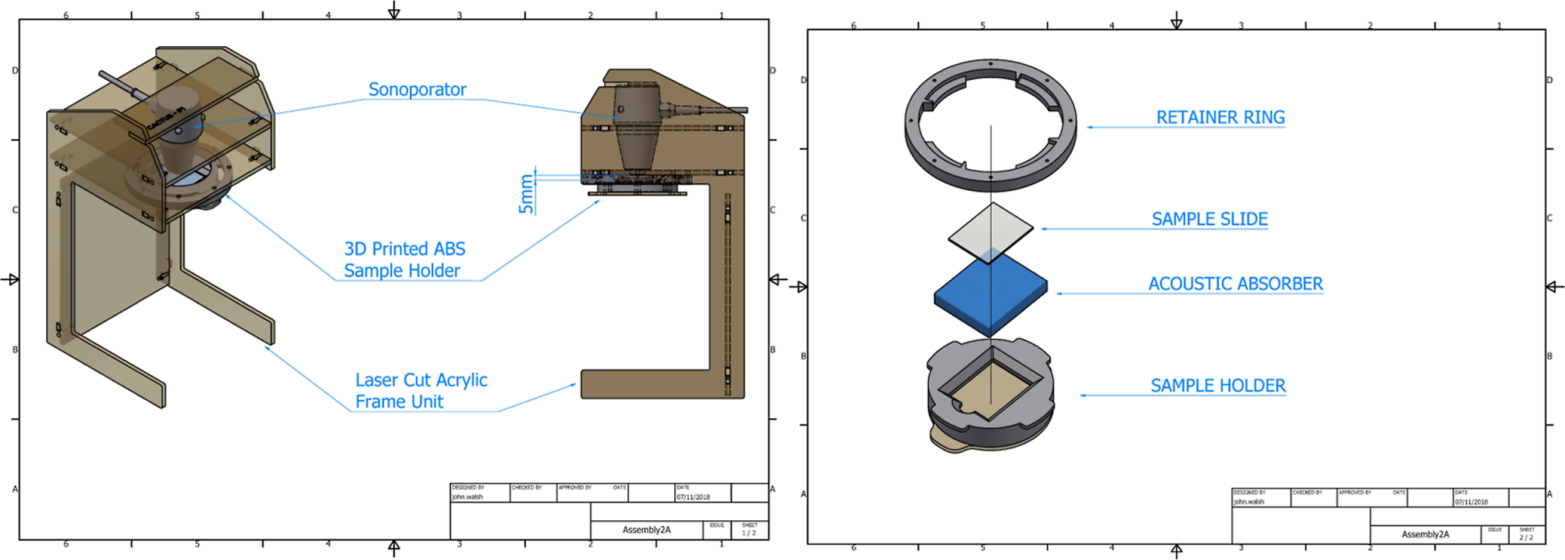
3D models of the main housing (left) and hologram/sample holder (right) sections of the prototype ultrasound testing unit.

The prototype ultrasound testing unit was designed using Autodesk Inventor software and fabricated from a combination of laser cut acrylic sheet and 3D printed acrylonitrile butadiene styrene components. The unit, shown in Figure 3, was designed through an iterative process using a combination of rapid prototyping equipment and processes including laser cutting and 3D printing. The main housing of the testing unit was made from laser cut acrylic which provided a structure into which the sonoporator transducer could be easily and firmly positioned. 3D printing was used to create a twist-in holder to accurately position the hologram/sample on the polyurethane-based acoustic absorber. Each component was designed specifically for this study and assembled into the bespoke finished unit. The result of this design process was a unit that ensured accurate repeatability of the testing process.

## 3 Results and Discussion

### 3.1 Hydrophone and Radiation Force Balance Measurements of the Sonoporation System

**Figure 4:**
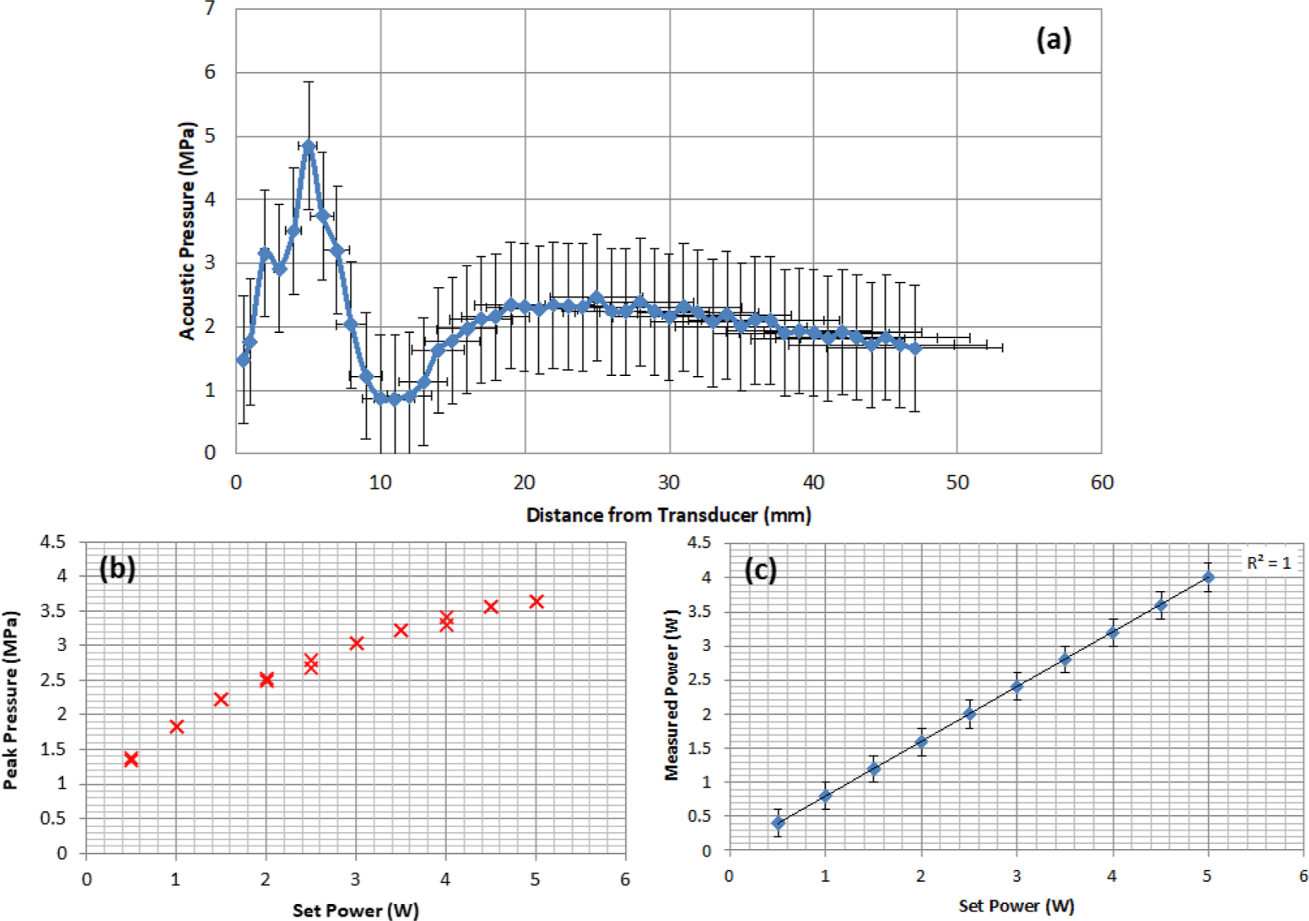
(a) Acoustic field plot of the spatial peak temporal average acoustic pressure along the center of the *z*-axis from the transducer face. (b) Peak pressure calibration as a function of increasing nominal set power at *z* = 5 mm from the face of the transducer. (c) Measured power as a function of set power at *z* = 5 mm.

For the sonoporator, the position of the region of maximum acoustic pressure in the *z*-direction needed to be determined for repeatable setup of the hologram measurement system in the initial calibration and subsequent experiments. The results for the acoustic field and pressure output characterization of the sonoporation system are shown in Figure 4. Figure 4a clearly shows that, for 100% duty cycle (i.e., continuous output), the acoustic field has a sharp peak at *z* = 5 mm from the transducer face. This *z* = 5 mm point corresponds to the point of the maximum pressure output for the SP100 transducer. Figure 4b shows that the peak pressure response at 100% duty cycle increases as a function of increasing nominal set power at *z* = 5 mm. Figure 4c shows the results from the radiation force balance measurements of acoustic power; the measured power increases linearly as a function of set power.

### 3.2 Calibration of Hologram Surface Temperature in Response to Ultrasound Exposure

**Figure 5:**
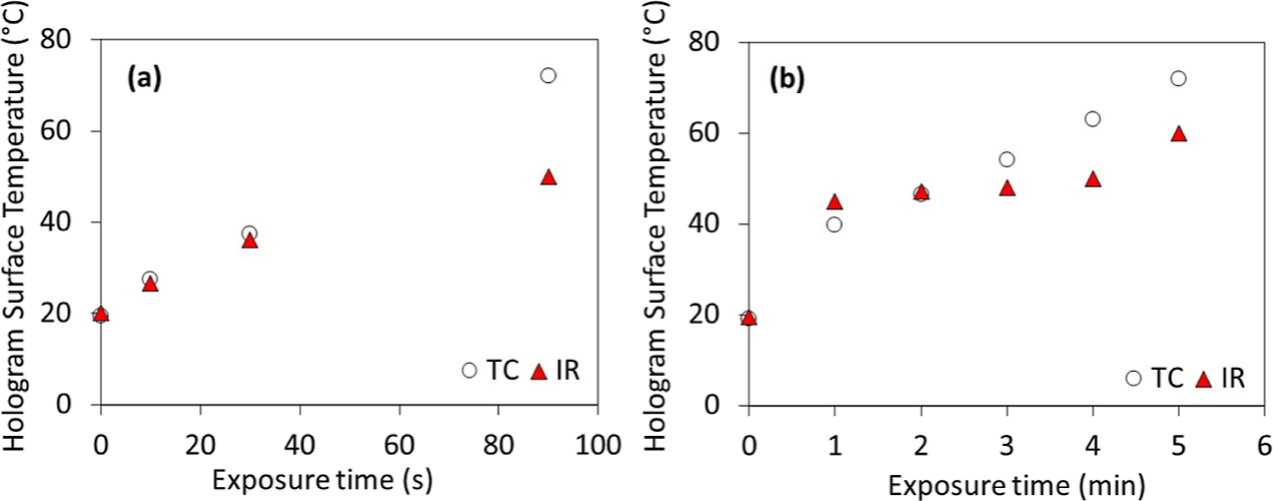
Hologram surface temperature (°C) vs exposure time measured via wire TC and infrared camera (IR) for the (a) sonoporation system (5 W/cm^2^) and (b) physiotherapy system (3 W/cm^2^).

The surface temperature of the holograms following ultrasound exposure by both the sonoporation and physiotherapy systems has been measured using the wire TC and the infrared camera methods, and the results are presented in Figure 5.

For both systems, the hologram surface temperature is observed to increase with exposure time. Due to the higher maximum output of the sonoporation system, the temperature increase occurs over a much shorter time duration. While the temperature values produced by the TC and infrared camera methods are in general agreement, there are some discrepancies, particularly at longer exposure times. For the longest exposure times tested on both the sonoporation (90 s) and physiotherapy (5 min) systems, the TC temperature values exceed the IR camera measurements by 40 and 16%, respectively. This is likely because the TC provides real-time in situ temperature data of the hologram surface, whereas the IR camera data are obtained following the removal of the transducer and, in the case of the sonoporation system, the coupling gel. The smaller discrepancy in the physiotherapy unit temperature values may be explained by the fact that the coupling medium is water, rather than ultrasonic gel, and so the IR camera images can be captured more quickly, minimizing losses due to dissipation of thermal energy into the environment. It is to be expected that the discrepancies between the TC and IR camera data are greatest for longer exposure times, due to the higher temperature rises achieved. Following the removal of the source of heat, the gained thermal energy will be quickly dissipated to the surrounding environment which is at room temperature. Following shorter exposure times, the temperature gradient relative to the surrounding environment is smaller and so energy loss will proceed at a slower rate. It should also be noted that while it is certainly possible to study the relative influence of increasing temperature using the TC method, the absolute temperature values obtained should be treated with caution, due to the possibility of the presence of viscous heating artefacts which can occur when metal TCs are used in an ultrasound field.^36^

### 3.3 Response of Holograms to Sonoporation Exposure

**Figure 6:**
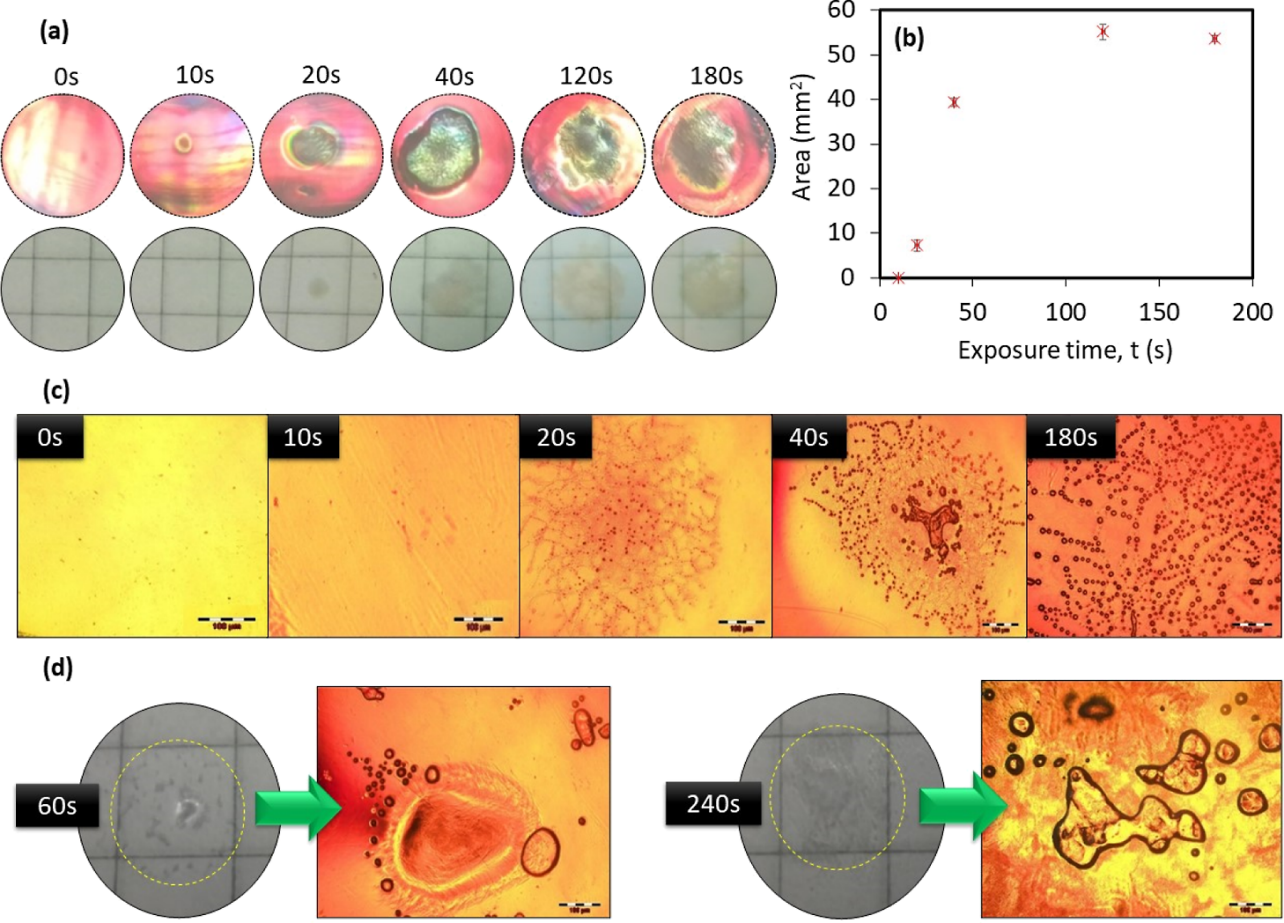
(a) Top row: photographs of reconstructed reflection holograms after 0–180 s of sonoporation exposure. Bottom row: photographs of hologram opaque structure on a 7 mm × 7 mm grid taken at normal incidence. (b) Area of opaque structure in mm^2^ vs ultrasound exposure time, t. The error bars represent the standard deviation of triplicate measurements of the area, for each data point. (c) Phase contrast microscopy images of holograms exposed with the Sonidel SP100 for 0, 10, 20, 40, and 180 s. (d) Photographs and phase contrast microscopy images of bleached photopolymer films (no gratings) exposed for 60 and 240 s. Scale bar of microscopy images is 100 μm.

The influence of increasing sonoporation exposure time on the holograms was investigated by exposing the holograms for 0, 10, 20, 40, 120, and 180 s. The ultrasound power density, pulse duty cycle, and pulse frequency were set at 5 W/cm^2^, 85%, and 100 Hz, respectively. The distance *z* was set at 5 ± 0.5 mm. A new hologram was used for each test. The results are shown in Figure 6. During ultrasound exposure, a visible opaque structure forms in the exposed area of the sample (Figure 6a). When illuminated with a white light source and viewed at an appropriate angle, this opaque region reconstructs green light. The area of the opaque region on each hologram was estimated as described in Section 2.3.3. The results shown in Figure 6b reveal that the size of the area experiencing structural changes with time increases until 120 s, after which it plateaus.

The influence of the distance, *z*, between the transducer face and the hologram on the ultrasound-induced reconstructed hologram color change was then investigated. The ultrasound power density, pulse duty cycle, and pulse frequency were again set at 5 W/cm^2^, 85%, and 100 Hz, respectively. The distance, *z*, was varied from 0 to 10 mm, and a longer exposure time of *t* = 240 s was used in order to ensure a response for larger *z* values away from the point of maximum acoustic pressure. The results (Figure S1) show that the maximum size of the color change/opaque area is obtained at *z* = 5 mm, whereas at other positions, practically no region of color change is observed. This indicates that, as expected, proximity to the point of maximum acoustic pressure influences the formation of the color change region and suggests that the hologram can be used as an alternative tool to the hydrophone to map the acoustic pressure profile of the ultrasound beam.

In order to determine the origin of the opaque/color change structure formed during sonoporator exposure, phase contrast microscopy images of the holograms before and after ultrasound exposure were obtained. It is clearly seen in Figure 6c that a structure, which appears to consist of small “bubbles”, forms with increasing exposure times. The area of the structure grows with increasing time, as does the size of the bubbles themselves. This is possibly due to neighboring bubbles merging. The same test was repeated in photopolymer layers which contain no holographic structure (Figure 6d). A similar opaque structure forms, and the phase contrast images show some large bubbles; however, the same structure observed in the hologram was absent.

**Figure 7:**
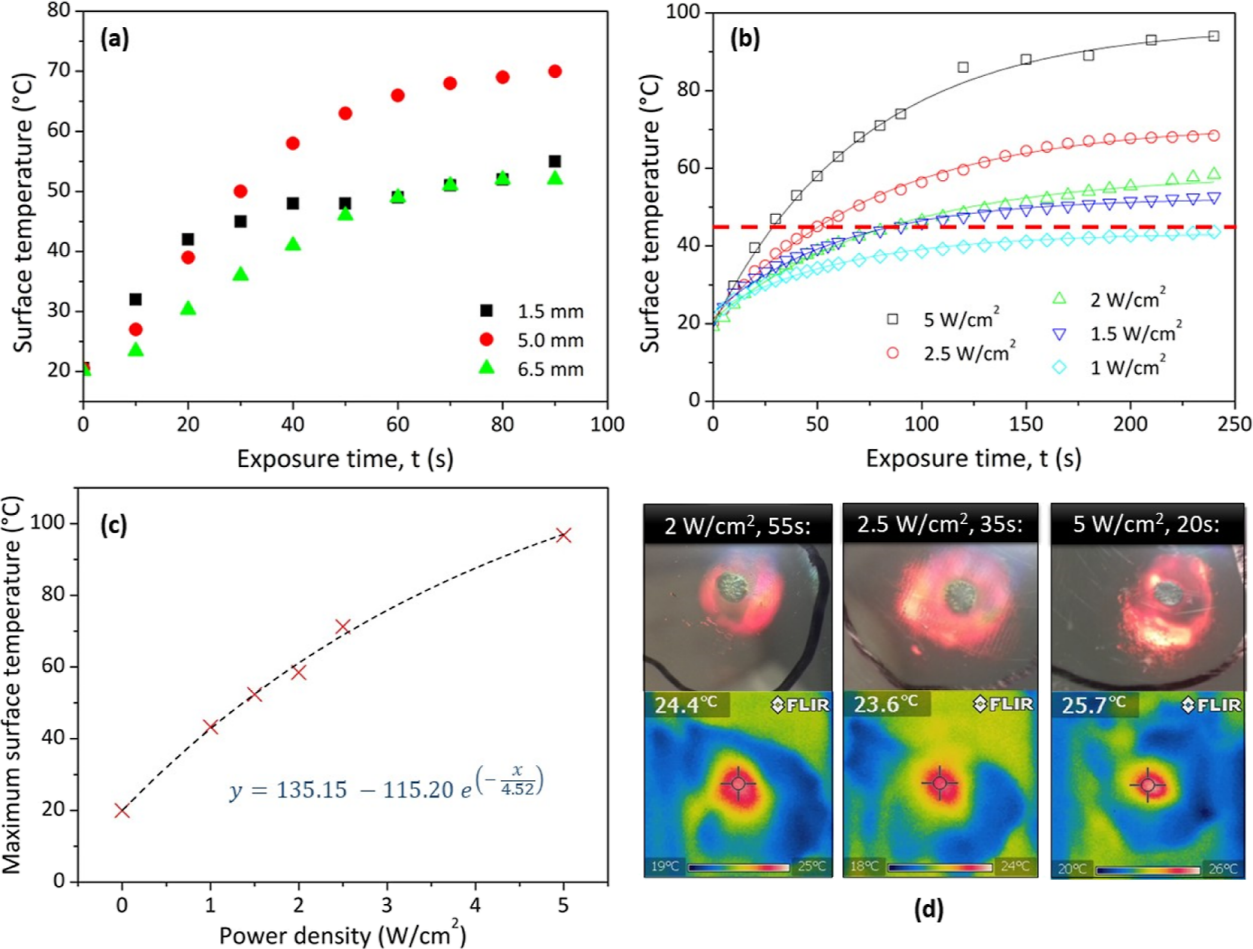
Surface temperature of the hologram measured via TC vs ultrasound exposure time, t for (a) different *z* values and (b) different power density values. The red dashed line in (b) indicates a threshold or switching temperature (i.e., temperature at which the formation of the color change region begins) of approximately 45 °C. (c) Maximum hologram surface temperature vs power density (W/cm^2^); the black dashed line is an exponential fit of the data (line equation shown). (d) Photographs and IR thermograms of three holograms exposed to 100 ± 15 J/cm^2^.

The role of temperature in the formation of the opaque/color change area on the holograms was then investigated. Figure 7a shows the surface temperature of the holograms obtained using a power density of 5 W/cm^2^ for exposure times of 0 to 90 s, at three different *z* values (1.5, 5.0, and 6.5 mm), measured via the wire TC method. All measurements were repeated three times. As expected, the temperature at the surface increases with increasing exposure time. The increase in temperature is largest at *z* = 5 mm, which is logical as this corresponds to the point of maximum acoustic pressure of the ultrasound beam.

The hologram surface temperature was then measured for different power density values. Figure 7b shows the temperature measured via TC vs exposure time for power densities of 1, 1.5, 2, 2.5, and 5 W/cm^2^. The red dashed line in Figure 7b represents a threshold or switching temperature of 40–45 °C, above which the formation of the irreversible opaque/green color change region is observed on the hologram surface. For the lowest power density of 1 W/cm^2^, no color change is obtained, even for significantly high exposure times of 240 s, as a surface temperature of 40–45 °C is never reached. Any combination of power density and exposure time will produce this ultrasound-induced response, provided that this threshold temperature is reached.

In order to extract further data regarding the rate of change of surface temperature with time, the data in Figure 7b were fitted using Origin v. 8.5 software with a single exponential decay function of the form , where *y*_0_ is the maximum/final temperature reached, *A* is an amplitude factor, and τ is a time constant. The results of the fit are presented in Table 3. The *R*^2^ values for each set of data show good agreement between the exponential fit and the experimental data.

**Table 3:** Results from Single Exponential Decay Fit of Surface Temperature vs Exposure Time Data

power density (W/cm^2^)	*y*_0_ (°C)	*A* ± Δ*A*	*T* ± Δτ (s^–1^)	*R*^2^ value
1.0	43.3	–20.3 ± 0.3	63.23 ± 2.79	0.993
1.5	52.4	–29.6 ± 0.4	61.90 ± 2.31	0.995
2.0	58.5	–37.4 ± 0.7	82.48 ± 4.94	0.991
2.5	71.3	–50.2 ± 0.4	78.51 ± 2.08	0.998
5	96.8	–76.9 ± 1.1	71.25 ± 2.85	0.997

As expected, the maximum temperature reached, *y*_0_, increases with both increasing exposure time and power density and follows a single exponential trend; the maximum surface temperature vs power density data shown in Figure 7c was well-fitted with the same single exponential decay function used previously, achieving an *R*^2^ value of 0.994. An interesting trend is observed in the τ data in Table 2. For the lowest tested power densities of 1.0 and 1.5 W/cm^2^, τ is the shortest, indicating that the fastest rate of change of surface temperature with time is obtained; this is counterintuitive. As the power density is increased to 2.0 W/m^2^, τ increases by just over 30%, implying that the rate of change of surface temperature with exposure time slows down. Then, as the power density is increased further to 2.5 and 5.0 W/cm^2^, the value of τ gradually decreases again, that is, the rate of change increases. It is hypothesised that the value of τ depends on the extent of formation of gas bubbles inside the hologram. For the two lowest power densities, 1.0 and 1.5 W/cm^2^, the gas bubbles were absent or minimal and so the incident ultrasound energy is readily absorbed by the polymer-based hologram, and it heats up quickly. As the power density increases to 2 W/cm^2^ and the hologram temperature increases correspondingly, the production and growth of gas bubbles resulted in the hologram heating more slowly, that is, τ becomes larger. This reduction in heating rate may be due to the ultrasound energy being dissipated through the bubble formation process or that the bubbles themselves impede the absorption of ultrasound energy inside the hologram through the scattering of the ultrasound wave. Further increase of the power density will supply sufficient energy to overcome the effect of the gas bubbles, and the rate of change of temperature will again increase, albeit at a slower rate than that observed in “bubble-free” holograms.

It can be deduced that the temperature at the hologram surface, which is directly related to the temperature in the volume of the film, is indicative of the extent of change in the hologram color which will occur due to insonation and depends on the delivered exposure energy, that is, the product of power density and exposure time. Figure 7d shows three different holograms where 100 ± 15 J/cm^2^ of ultrasound energy has been delivered using different combinations of power density and exposure time. In each case, the same approximate size color change region is obtained. This further highlights the need for operators to consider the combined influence of both the treatment acoustic pressure (set by the power density) and the treatment exposure time.

Further data consisting of photographs, IR thermograms, and phase contrast microscopy images for holograms exposed for 20, 40, and 90 s at *z* = 5 mm are included in Figure S2. The size of the opaque/color change area, the IR surface temperature, and the size of the bubbles are all seen to increase as the exposure time is increased from 20 to 90 s. This was repeated for other *z* values (1.5 mm—Figure S3, 6.5 mm—Figure S4), and the same trends were observed.

Temperature elevation alone is insufficient to produce the color change/opaque region in the photopolymer-based holograms. Extended thermal-only treatment of samples at temperatures in excess of 100 °C produces no such change. Therefore, it is postulated that the color change/opaque region in the holograms was achieved due to the temperature-assisted (>40 °C) modification of the polymer film combined with the delivery of acoustic pressure. Ultrasonic degradation of polymers such as polyacrylamide (PAA) is reported in the literature;^37,38^ “degradation” in this context is defined as a change in the chemical and/or physical structure of the polymer chain, which causes a decrease in the molecular weight of the polymer. The opaque region is therefore likely visible due to the modification of the diacetone acrylamide and bisacrylamide polymer chains within the photopolymer film; the accompanying visible green color is likely due to the distortion of the reflection grating and its planes by the generated bubbles. This distorted grating likely reconstructs at multiple wavelengths, but the sum is interpreted by the eye as green.

### 3.4 Response of Holograms to Diverging Beam Physiotherapy Exposure

**Figure 8:**
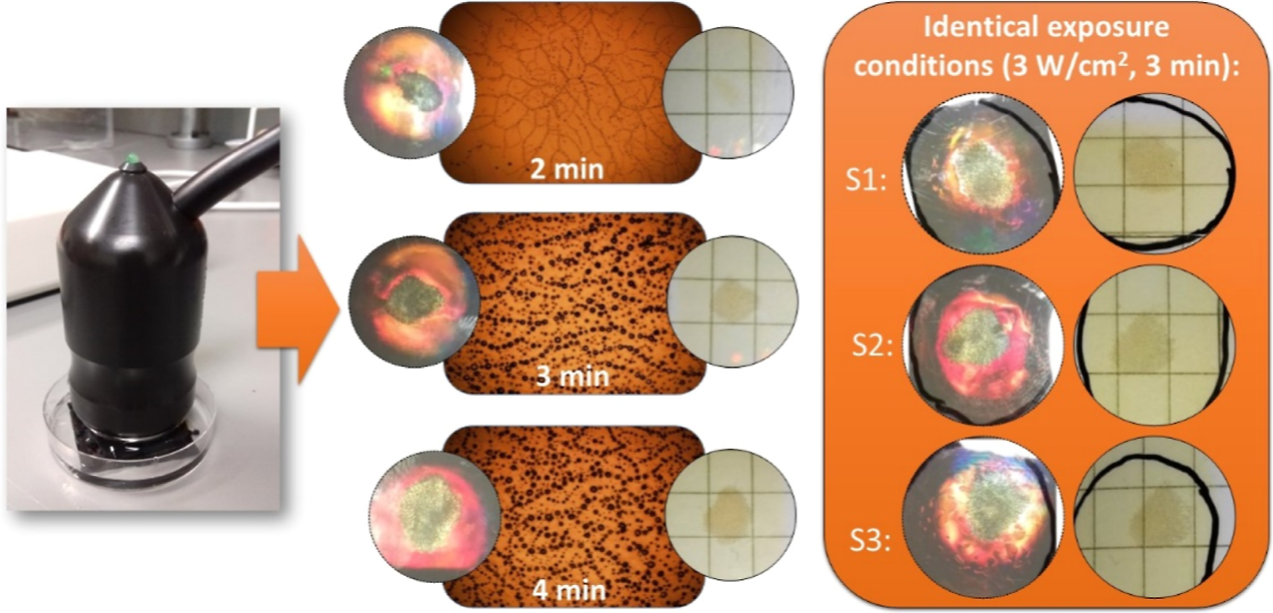
Phase contrast microscopy images and photographs of holograms following 2, 3, and 4 min exposure from the physiotherapy transducer shown. Far right: photographs of three identical devices following identical exposures (3 W/cm^2^, *t* = 3 min).

A study of the response of the holograms to exposure by the diverging ultrasound beam produced by the physiotherapy system was conducted, as described in Section 2.2.2. A new hologram was used for each measurement. Photographs and phase contrast microscopy images of the reconstructed holograms following 2, 3, and 4 min exposures are shown in Figure 8. Unlike the sonoporator system, the threshold exposure time via the diverging beam system is 2 min, below which no response is observed. Like the sonoporation studies, an opaque/color change region is observed to form, the size of which increases with increasing exposure time. The size of the ultrasound-induced gas bubbles seen in the phase microscopy images is again observed to increase. As discussed in Section 3.3, this is possibly due to the increased extent of temperature-assisted ultrasonic degradation inside the photopolymer film. To verify repeatability of the response, three identical but separate holograms were exposed for 3 min to an ultrasound power density of 3 W/cm^2^. The results are shown in Figure 8. For identical exposure conditions, the measured area of the opaque/color change region produced was highly repeatable across the three samples (50.0, 49.9, and 47.1 mm^2^) with a standard deviation of less than 3.5%.

### 3.5 Calibration Curves for the Sonoporation and Physiotherapy Systems

**Figure 9:**
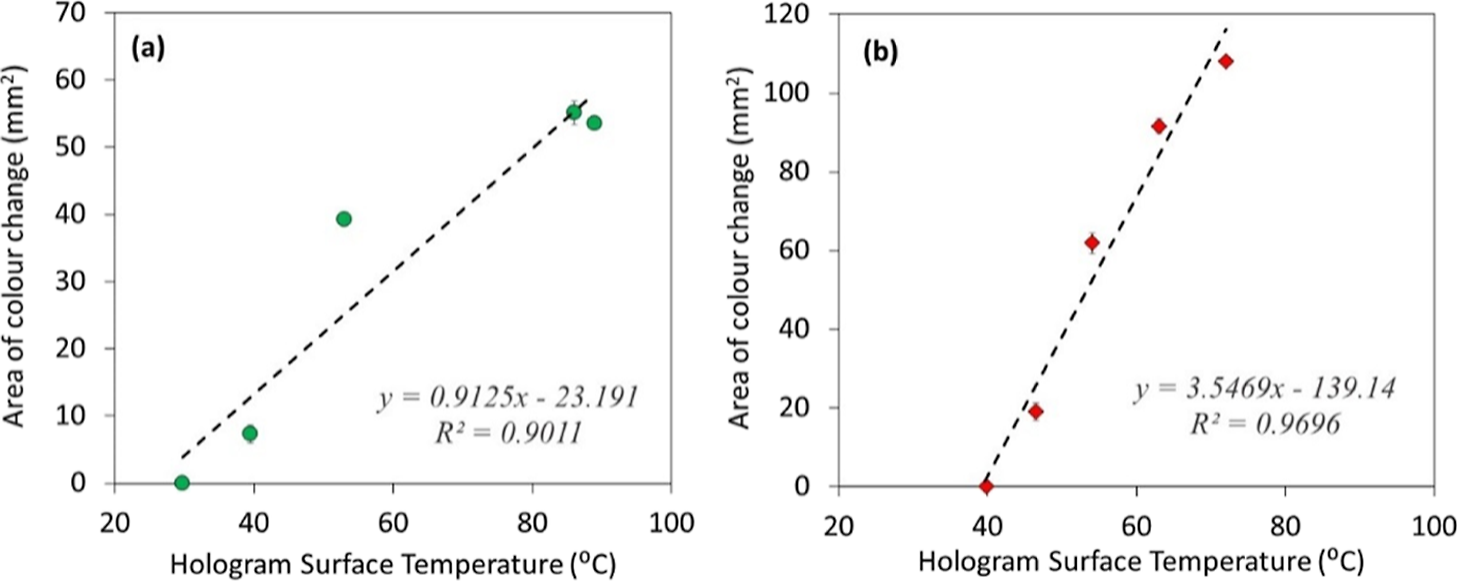
Area of color change (mm^2^) vs hologram surface temperature (°C, measured via wire TC) for the (a) Sonidel sonoporation system and (b) gbo physiotherapy system. The black dashed line is a linear fit of the data (line equation and *R*^2^ value shown).

Calibration curves for the expected area of hologram color change produced by ultrasound exposure from both the sonoporation and physiotherapy systems are presented in Figure 9. Due to the differences in the maximum output power produced by the two systems, the absolute values of color change area are distinct. However, for both systems, the area of the color change region increases linearly with increasing temperature (which itself is due to increasing exposure time as per Figure 5). It is expected that for any ultrasound system, the produced hologram surface temperature and thus the hologram color change area will be consistent over time, once the ultrasound system output is itself consistent. Therefore, this data serves as a calibration curve for each specific ultrasound system that must be collected as part of the initial establishment of a QA testing protocol, which then can be used to monitor the output of the ultrasound system over time. Any diminishment in the hologram color change area during routine QA testing will indicate a reduction in hologram surface temperature and thus an imperceptible change in the ultrasound system output.

It has also been seen that the threshold exposure conditions required to produce the minimum area of color change also depend entirely on the ultrasound system beam profile. For the polymer-based hologram, the threshold exposure conditions will correlate roughly with a hologram surface temperature of 45 °C. The exposure conditions required to achieve this temperature will vary between systems; for example, for the Sonidel sonoporator, 20 s exposure at 5 W/cm^2^ is sufficient, whereas for the gbo physiotherapy unit, 2 min at 3 W/cm^2^ is required.

### 3.6 Prototype Ultrasound Testing Unit

**Figure 10:**
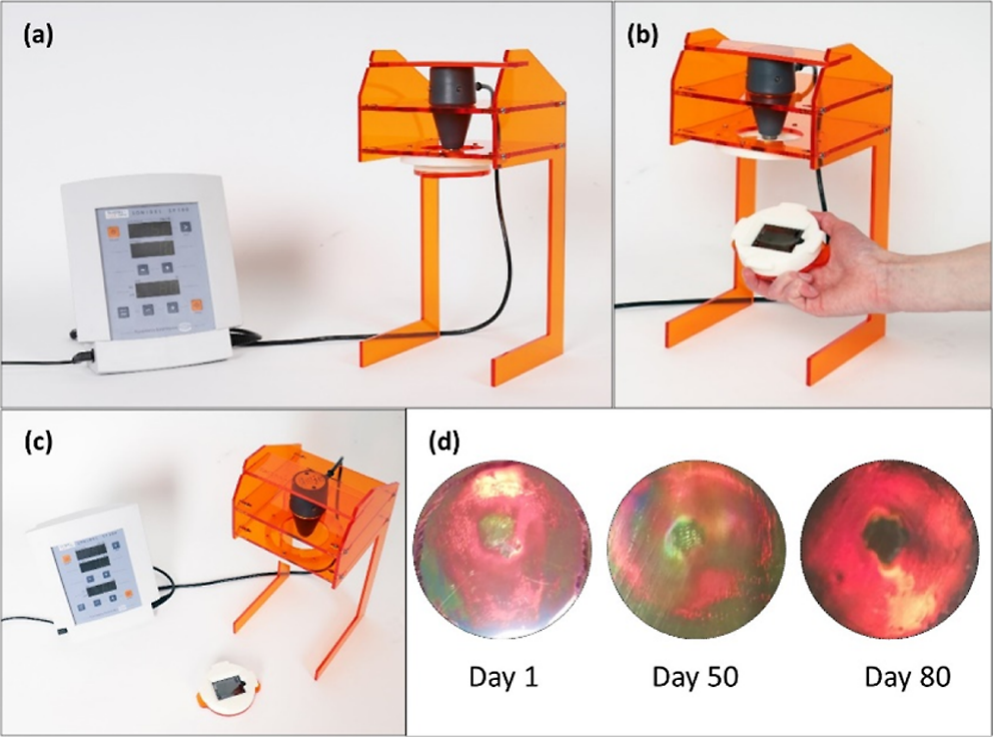
(a–c) Photographs of the prototype ultrasound testing unit. (d) Photographs of three identical holograms tested in the prototype unit 1, 50, and 80 days after their fabrication (*t* = 60 s; power density = 5 W/m^2^; duty cycle = 85%).

The assembled prototype ultrasound testing unit is shown in Figure 10a–c. The SP100 Sonidel transducer is slotted into the top of the unit in a fixed position and is held securely in place by a further plastic retainer. The hologram is placed into a custom build holder which contains an acoustic absorber backing material to absorb the transmitted ultrasound energy. The holder is then easily screwed (quarter turn) into the underside of the testing unit into a fixed position at *z* = 5 cm from the face of the transducer. The hologram can be quickly changed without removing the transducer. The reproducibility over time of the holograms’ responses when tested in the testing unit has been verified (Figure 10d). In addition, the photopolymer-based holograms themselves are stable and maintain their bright red color over a minimum period of 80 days, once laminated. It is envisaged that in its current form, the prototype testing unit will facilitate periodic testing of the transducer output with the holograms, on a weekly or monthly basis, as required. If the transducer is performing as expected, the same size region of color change will be observed on the hologram surface with each test, for identical Sonidel SP100 output settings. Ideally, positioning of the hologram at a variety of depths along the *z* axis of ultrasound beam would also be possible, as this would allow 3D beam profile to be mapped. An adjustable height device holder will be incorporated into the next iteration of the prototype to accommodate this.

## 4 Conclusions

A hologram which is capable of qualitatively assessing the output of therapeutic ultrasound systems has been reported. The hologram, consisting of a reflection grating fabricated in a diacetone acrylamide-based photopolymer, produces a visible change in the color of the reconstructed hologram from red to green in response to incident ultrasound energy, at an approximate switching temperature of 45 °C measured at the hologram surface. This response has been verified for two different therapeutic ultrasound systems; this demonstrates the wide applicability of the technique. Studies with the sonoporation system verified that the size of the color change region is dependent on the ultrasound-induced temperature increase in the film, which is in turn governed by the ultrasound system acoustic output and exposure time; temperature alone is insufficient to produce a change in color. The size of the color change region can be readily quantified, either visibly or using open source image analysis software such as ImageJ and compared to a reference measurement obtained during initial commission of the ultrasound system. If the system is functioning as intended, this color change region should remain constant over time. The hologram response is quick and reproducible. Combined with the described prototype ultrasound testing unit, the holograms offer a fast, straightforward benchtop QA approach which can be easily adopted by ultrasound operators in a busy clinical setting with minimal training for pre-treatment checks and routine QA. A short video demonstrating the concept and operation of the hologram as part of routine therapeutic ultrasound physiotherapy QA was developed and is available to watch as Supporting Information to this paper and online.^39^

Future work will include trialing of the holographic QA devices in a clinical physiotherapy setting, as well as design and fabrication of the next iteration of the prototype testing unit. A further application of this prototype ultrasound testing unit is the integration into the daily QA of MR-guided FUS or HIFU systems prior to patient treatment. Both polyvinyl alcohol-based thermochromic materials^40^ and PAA-based tissue-mimicking phantoms^41−43^ have previously been reported in the literature as suitable for HIFU treatment planning, ablation visualization, and verification. However, these materials require a higher switching temperature in order to align with the required HIFU threshold temperature of 56 °C compared to the lower switching temperature of 45 °C of our device. Future work will include investigation of the ability to tune the switching temperature of the photopolymer-based holographic ultrasound sensor through modification of the photopolymer composition via the use of alternative monomers and variation of the crosslinker concentration.

## Supporting Information Available

Demonstration of the operation of the holographic sensor (MP4)
Graphed data of sonoporator output (*V*^2^) vs hydrophone distance, *z*, from the transducer (mm), overlaid with photographs of hologram color change; photographs, IR thermograms, and phase contrast microscopy images for holograms exposed for 20, 40, and 90 s at *z* = 5.0 mm; photographs, IR thermograms, and phase contrast microscopy images for holograms exposed for 20, 40, and 90 s at *z* = 1.5 mm; photographs, IR thermograms, and phase contrast microscopy images for holograms exposed for 20, 40, and 90 s at *z* = 6.5 mm. (PDF)
