# Supplementary material for: Color-Changing Reflection Hologram for Quality Assurance of Therapeutic Ultrasound Systems
Source: ACS Appl Mater Interfaces. 2023 Jul 21;15(30):36792–803. doi: 10.1021/acsami.3c06139 (PMC10401507; doi:10.1021/acsami.3c06139)
Supplement: Supplementary file 2 — am3c06139_si_002.pdf [file am3c06139_si_002.pdf]

SUPPORTING INFORMATION

A colour-changing reflection hologram for quality assurance of therapeutic ultrasound systems

Tatsiana Mikulchyk,[1] John Walsh,[2] Jacinta Browne,[1,3] Izabela Naydenova,[1] Dervil Cody[1,]*

[1]Centre for Industrial and Engineering Optics, School of Physics, Clinical and Optometric Sciences, Technological University Dublin, Grangegorman Campus, Central Quad, Grangegorman Lower, D07 ADY7, Dublin, Ireland.

[2]School of Art and Design, Technological University Dublin, Grangegorman Campus, Grangegorman Lower, D07 ADY7, Dublin, Ireland.

[3]Department of Radiology, Mayo Clinic, Rochester, Minnesota, MN 55905, USA.

*dervil.cody@tudublin.ie

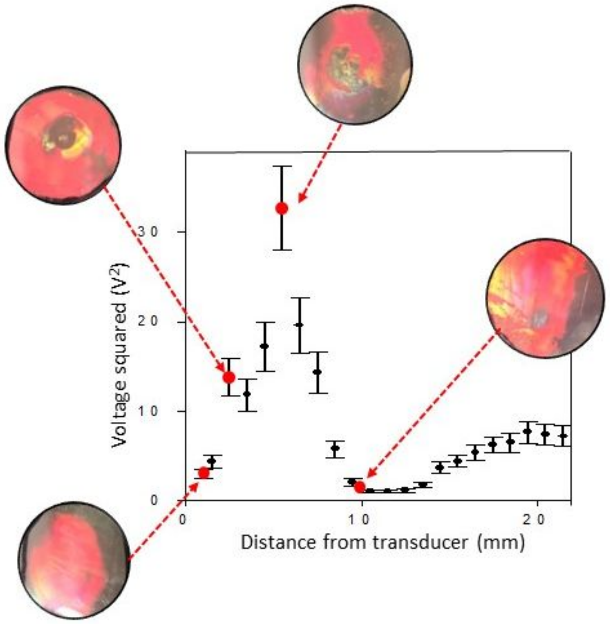

Figure S1. Graphed data shows sonoporator output (measured via hydrophone, units of voltage squared, V^2) vs. hydrophone distance, z, from the transducer (mm). Overlaid photographs show the hologram colour change observed due to sonoporation exposure when the hologram is positioned at the corresponding z distances from the transducer.

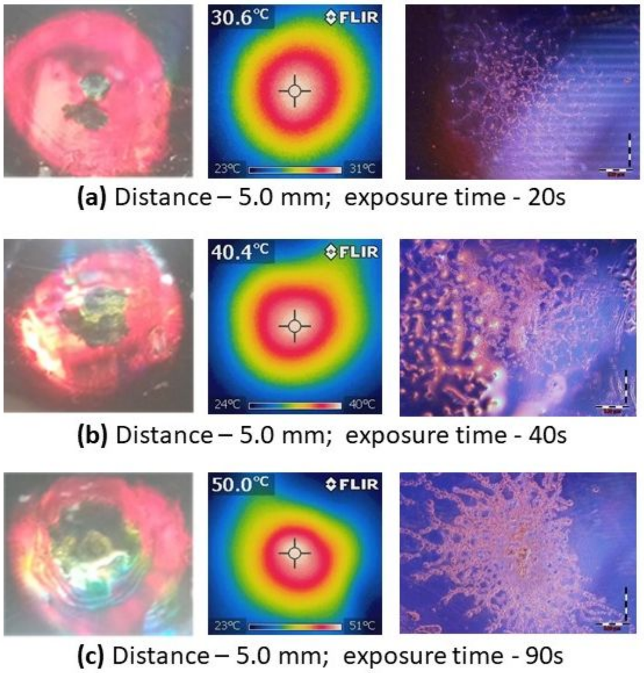

Figure S2. Data collected following ultrasound exposure of holograms located at 5.0 mm from transducer face for exposure times of (a) 20s, (b) 40s and (c) 90s. From left to right: photograph of the image produced by the hologram under ambient light illumination after ultrasound exposure; IR thermogram of the layer immediately after ultrasound exposure; phase contrast microscope image of the layer surface after ultrasound exposure.

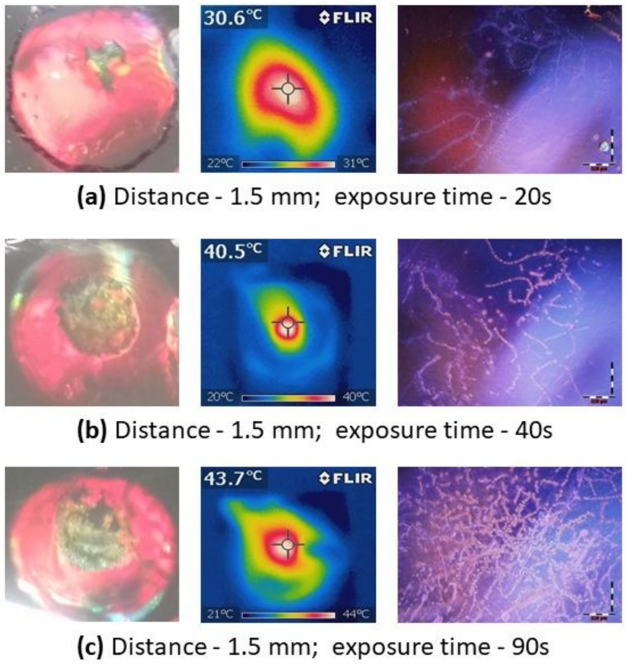

Figure S3. Data collected following ultrasound exposure of holograms located at 1.5 mm from transducer face for exposure times of (a) 20s, (b) 40s and (c) 90s. From left to right: photograph of the image produced by the hologram under ambient light illumination after ultrasound exposure; IR thermogram of the layer immediately after ultrasound exposure; phase contrast microscope image of the layer surface after ultrasound exposure.

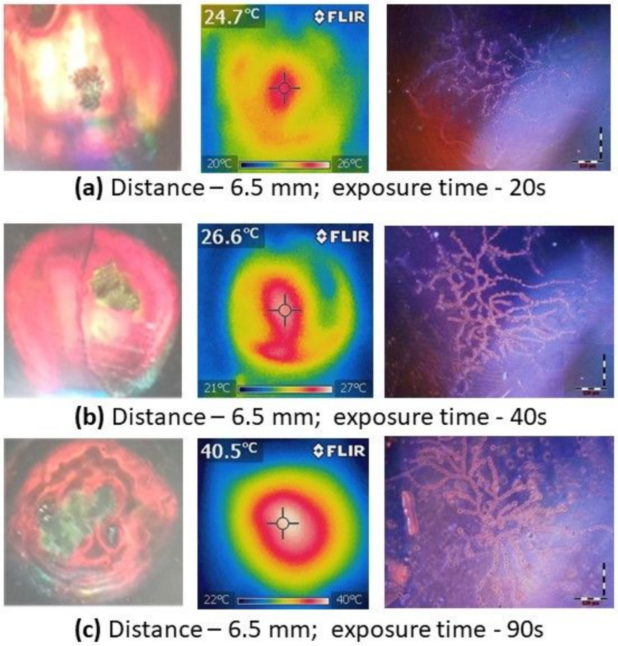

Figure S4. Data collected following ultrasound exposure of holograms located at 6.5 mm from transducer face for exposure times of (a) 20s, (b) 40s and (c) 90s. From left to right: photograph of the image produced by the hologram under ambient light illumination after ultrasound exposure; IR thermogram of the layer immediately after ultrasound exposure; phase contrast microscope image of the layer surface after ultrasound exposure.
